# Lanolin

**Published:** 1886-04

**Authors:** 


### Lanolin.

Lanolin, a cholesterine fat derived from wool, is a new basis for ointments and pomades proposed by Liebreich. It is a viscid, oily substance, the viscidity being its chief objection, on account of which it becomes necessary to add other substances, such as vaseline, parafine, glycerine, oils or fats. But it has certain advantages which more than counterbalances these objections:

1. It is easily absorbed by the skin. Liebreich has found that the fats derived from keratin-bearing tissues are most readily absorbed by the skin.

2. It does not produce irritation. Lassar has employed it in four hundred cases of skin disease, and has never observed any irritating properties. For this reason it is to be highly recommended in massage.

3. It is readily miscible with water and glycerine, thus reducing it to any desired consistency. Yet, when the pores of the skin have been filled with lanolin, although the skin is not greasy, it will not absorb water. For this reason it is to be recommended to physicians who are obliged to handle septic matters.

4. It is miscible with mercury, taking up this metal in a remarkable manner, and forming a perfectly homogeneous mass. No globules of the metal can be detected, even with the miscroscope. For the treatment of syphilis by inunctions, it offers very great advantages.

5. It absorbs its own weight of alkaline and neutral salts, thus making it of the greatest value to dermatologists. Lanolin is exceedingly useful in the treatment of *seborrhea sicca*, pityriasis, and chapped and fissured skin. As a basis for a sulphur ointment, it gives admirable results in pruritus ani. A twenty-five per cent. lanolin-chrysarobin ointment cures psoriasis very rapidly, without producing irritation.

The demand for lanolin has already become so great, that other substances, sold under the name of wool-fat, are being placed upon the market. Many of them are injurious, as they contain free acids which injure the skin. Liebreich suggests the following tests for its purity, which we quote in full from the *Therapeutic Gazette:*

1. A small quantity, on being heated in water over a water-bath, must show the absence of glycerine.

2. If a solution of caustic soda be added, ammonia must not be developed.

3. The fat, if a small portion be heated with water on a water-bath, must separate in oily drops without producing an emulsion. If the quantity employed be large, it must separate as a clear oil.

4. With blue litmus-paper the reaction must not be acid.

5. For examination, if it be mixed or well rubbed with water upon a ground-glass plate with an iron spatula, the result must be a product containing over one hundred per cent. of water; and if the lanolin employed be pure, the kneaded mass will be sticky and paste-like, to which the spatula readily adheres; but if impure, the mass will have a soap-like smoothness, from which the spatula readily glides.

6. On exposure, the upper surface of lanolin and all lanolin salves and ointments become darkened, due to the evaporation of water, and not to its decomposition.

7. It never becomes rancid, and its smell should remind one of wool.

